# Sulphonylureas as Adjunct Therapeutic Agents in the Treatment of Autoimmune Conditions: A Narrative Review

**DOI:** 10.1002/prp2.70155

**Published:** 2025-07-21

**Authors:** Jasen Elford, Robert J. O'Reilly, Anna‐Marie Babey, Rukshan Ahamed Mohamed Rafeek, Natkunam Ketheesan

**Affiliations:** ^1^ School of Science & Technology University of New England Armidale New South Wales Australia

## Abstract

A rapid and cost‐effective arm of the drug discovery and development process is finding new uses for existing drugs. Initially used as antibacterial agents, sulphonylureas were repurposed for the treatment of type 2 diabetes due to their hypoglycemic side effects. Their primary mechanism of action is mediated by binding to sulphonylurea receptors (SUR), which are atypical adenosine triphosphate binding cassette transporters in pancreatic beta cells. This interaction inhibits ATP‐sensitive potassium channels to promote insulin release. Off‐target actions of sulphonylureas identified in recent studies have demonstrated a range of anti‐inflammatory properties mediated by modulation of the nucleotide‐binding oligomerization domain‐like receptor pyrin domain‐containing protein 3 inflammasomes. Inflammasomes are cytosolic protein complexes that assemble in response to infection or stress‐associated stimuli, activating inflammatory responses, and are the primary source of pro‐inflammatory cytokines. Sulphonylureas and their derivatives have been shown to inhibit various stages of inflammasome activation, leading to the reduction of pro‐inflammatory mediators, including IL‐1β and IL‐18. Recent evidence demonstrates that these agents reduced inflammatory responses, disease severity, and progression in various preclinical autoimmune and inflammatory models. In this narrative review, we consider the complexity of autoimmune conditions and the limited treatment options, and highlight the potential value of repurposing sulphonylureas and their derivatives as adjunct therapeutics for autoimmune conditions.

## Graphical abstract

NLRP3 Inflammasome activation drives inflammatory responses through the production and activation of pro‐inflammatory cytokines. Repurposing sulphonylureas and their derivatives to target NLRP3 may act as adjunct therapeutics for autoimmune conditions.

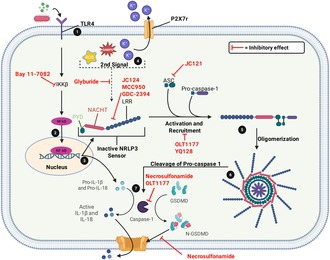

## 1 Introduction

The paucity of treatment options makes living with one or more than 100 autoimmune conditions a daily challenge for more than 3% of the global population [1]. Unfortunately, conventional drug discovery and development pathways generally require several decades for new therapies to become available for clinical use. By contrast, repurposing existing drugs by taking advantage of off‐target actions offers a potentially viable alternative. Investigations into these mechanisms offer valuable insights into novel uses of existing agents, many of which supersede their original purpose. One such drug class is the sulphonylureas.

Sulphonylureas have been used successfully for almost 50 years in conjunction with metformin in the treatment of type‐2 diabetes (T2DM). Their primary mechanism targets the sulphonylurea receptor 1 (SUR1), an atypical adenosine triphosphate (ATP) binding cassette (ABC) protein associated with the inwardly rectifying ATP‐dependent potassium (Kir6.2) channels on pancreatic beta cells [2, 3, 4]. Binding of sulphonylureas to SUR1 blocks these channels to prevent the efflux of potassium ions, depolarising the cell, leading to the activation of voltage‐gated calcium channels (VGCCs), which causes vesicle mobilization and release of insulin [2, 5]. Broadly, sulphonylureas are classified as either first‐ or second‐generation drugs, with second‐generation agents (gliclazide, glipizide, gliquidone, and glibenclamide) exhibiting greater potency [6]. However, intragenerational structural differences alter SUR subtype specificity, which underpin effects such as insulin secretion and ischaemic preconditioning [6].

Although the primary mechanisms are well‐defined, sulphonylureas and their derivatives exert a wide range of anti‐inflammatory effects [5]. Physiological outcomes include the reduction in high blood glucose levels, associated with increased levels of innate inflammatory markers, and the decreased risk of cardiovascular disease, neuropathy, and retinopathy [7, 8]. The anti‐inflammatory effects of sulphonylureas may be attributed to their ability to modulate the function of the nucleotide‐binding oligomerisation domain (NOD)‐like receptor pyrin domain‐containing protein 3 (NLRP3) inflammasomes [9]. Activation and release of the proinflammatory cytokines interleukin (IL)‐1β and IL‐18 mediated by the NLRP3 inflammasome cause cellular damage, leukocyte recruitment and tissue infiltration in several inflammatory and autoimmune diseases, including inflammatory bowel diseases (IBD), multiple sclerosis (MS), and rheumatoid arthritis (RA) [9, 10, 11]. Consequently, sulphonylureas targeting the NLRP3 inflammasomes may impair disease progression by limiting pro‐inflammatory cytokine activities, including exacerbating damage caused by autoantibodies and autoreactive T cells [12, 13].

Currently, treatment strategies for autoimmune diseases focus on immunomodulatory and antigen‐specific therapies to limit the direct immune‐mediated damage and lessen the subsequent inflammatory responses [14]. Since the sulphonylureas and their derivatives target several points within the inflammatory cascade, they could potentially complement current treatments to further modulate the immune response [14].

This narrative review evaluates the current targets of sulphonylureas and their derivatives, providing insights into their potential as adjunct therapies in the treatment of autoimmune conditions. A comprehensive review and critical analysis of published research sourced from PubMed and Google Scholar was undertaken to evaluate the targeted activity and efficacy of the sulphonylureas against the NLRP3 inflammasome, particularly in relation to a subset of related autoimmune conditions. The information gathered was then used to highlight the potential for these agents to target disease development in several models of autoimmune conditions.

## 2 Sulphonylurea Structures and Activity

### 2.1 Key Structures and Biological Targets of the Sulphonylureas

**FIGURE 1:**
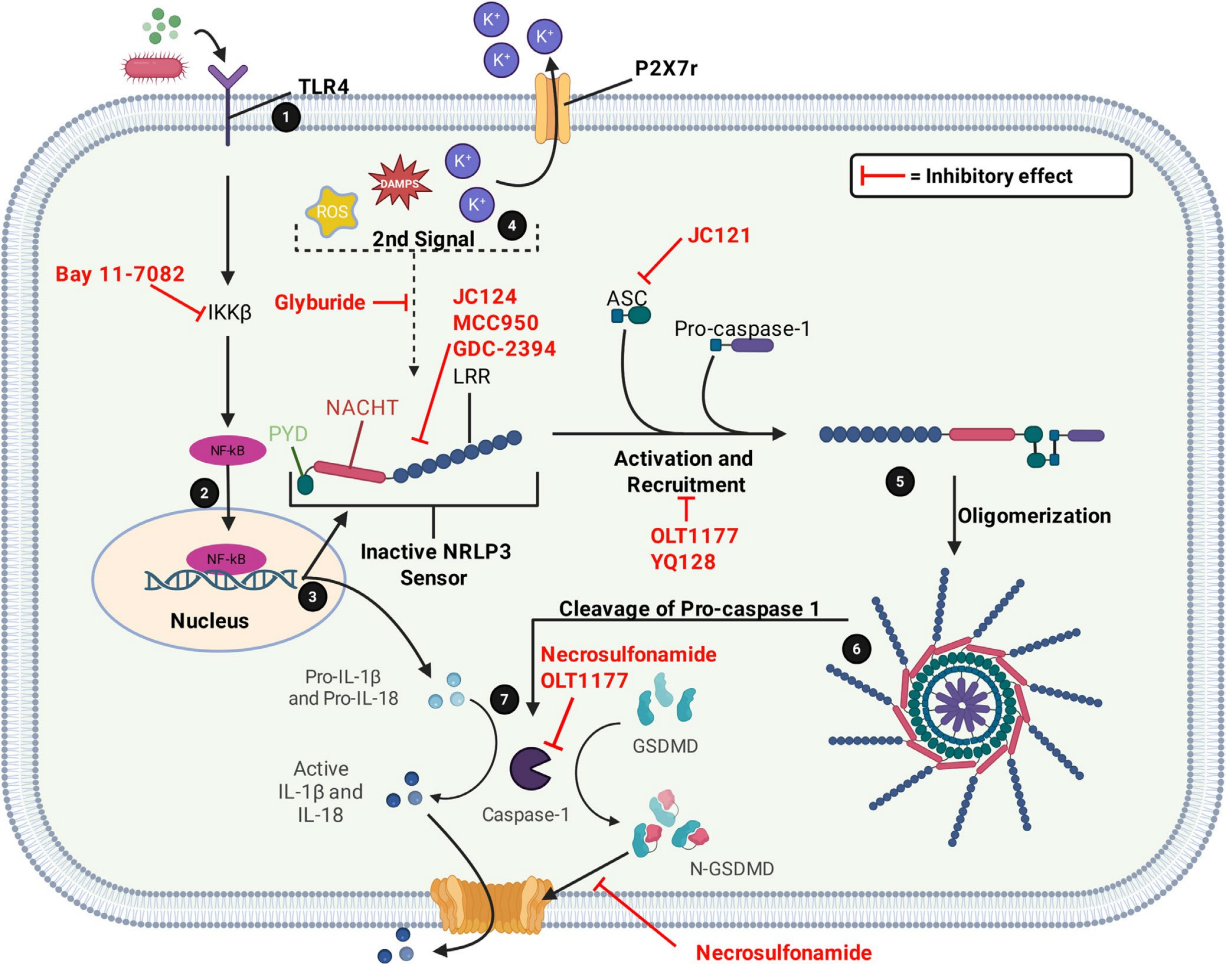
Signaling cascade and regulation points of the NLRP3 inflammasome. Inflammasome upregulation in monocytes, macrophages, and neutrophils occurs when TLR4 receptors are activated (1) causing translocation of NF‐κB (2), increasing production of the NLRP3 sensor, pro‐IL‐1β and pro‐IL‐18 (3). The second signal caused by DAMPs, ROS, and low intracellular potassium (K^+^) (4) activates the NLRP3 sensor, triggering recruitment and oligomerisation (5). Auto‐cleavage of the pro‐caspase‐1 region (6) results in its activation and the subsequent activation of IL‐1β, IL‐18, and the pore‐forming GSDMD (7). ASC, apoptosis‐associated speck‐like protein containing a caspase recruitment domain; DAMPs, danger associated molecular pattern; GSDMD, gasdermin D; IKKβ, inhibitor of nuclear factor kappa B kinase subunit β; IL, interleukin; LRR, leucine‐rich repeat; NACHT, central nucleotide‐binding domain; NF‐κB, nuclear factor kappa B; NLRP3, nucleotide‐binding domain, leucine‐rich–containing family, pyrin domain–containing‐3; P2X7, P2X purinoceptor 7; PYD, pyrin domain; ROS, reactive oxygen species; TLR4, toll‐like receptor‐4. Adapted from [9, 17, 18] (Created with biorender.com).

The structure of a sulphonylurea is comprised of two core constituents, a sulphonyl group and a urea group (Table 1) [3, 4]. Primarily, sulphonylureas stimulate insulin release by binding to SUR1.Kir6 channels present in the pancreatic beta cells [15, 16]. However, modification of the substituents attached at two positions within these compounds, namely the site *para* to the arylsulphonyl moiety (i.e., R^1^), as well as the nitrogen atom of the urea moiety (i.e., R^2^), is responsible for intragenerational variability in pharmacological activity (Table 1) [6]. These modifications alter the specificity and affinity for SUR subtypes, along with the duration of action, hypoglycaemic risk, and potential anti‐inflammatory effects [3, 4, 6]. Primarily, modification to R^2^ produces inhibitory effects against the NLRP3 inflammasome regulatory points (Figure 1) [11]. However, the precise nature of the R^1^ substituent of glibenclamide is critical for dual interaction with both SUR1 and the NLRP3 inflammasome [11].

**TABLE 1:** Chemical structures of various clinically significant first‐ and second‐Generation Sulfonylureas.

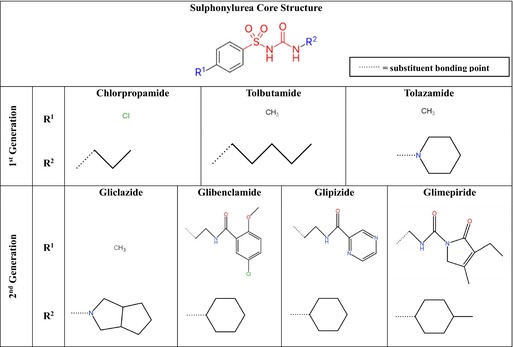

*Note:* The structures were adapted from [4].

The sulphonylurea off‐target interactions with the NLRP3 inflammasomes have prompted further investigations into their potential for treating acute and chronic inflammatory conditions [10, 19, 20, 21]. Insulin stimulation with the first‐ and second‐generation sulphonylureas requires a half‐maximal concentration range between 0.7 and 140 nM, with inhibition of IL‐1β requiring between 6.4 and 154.5 μM [22, 23]. Therefore, the current generations of sulphonylureas may pose a hypoglycaemic risk. However, doses of glibenclamide (20 mg/kg/day) have demonstrated an ability to prevent cardiac hypertrophy and reduce oxidative stress in mouse models via inhibition of the NLRP3 inflammasome pathway, without affecting blood glucose levels [24]. Furthermore, glibenclamide at doses of 0.001–1 nM has also been shown to improve retinal function through SUR1 binding [25]. In light of the potential role of sulphonylureas as immunomodulators, studies have focused on structure–activity relationship optimization to limit pancreatic interactions and improve the specificity towards the NLRP3 inflammasome [22, 23].

### 2.2 Sulphonyl‐Based Derivatives

Several derivatives of sulphonylureas have been synthesized that demonstrate reduced SUR1 interaction and promote NLRP3 specificity, increasing their anti‐inflammatory effects (Table 2) [10, 19, 20]. However, the relative effectiveness of chemically distinct sulphonylurea derivatives appears to be correlated with their affinity for, and inhibitory effects at specific regulatory points within the inflammasome signaling cascade (Figure 1). The sulphonylurea derivative MCC950 is the most efficacious NLRP3 inhibitor, with a half‐maximal concentration of 7.5 nM, and has been widely utilized across several preclinical models (Table 2) [10, 29]. Further alterations of glibenclamide and MCC950 have caused significant shifts in potency at, and specificity for SUR1 or the NLRP3 inflammasome, as well as functional duality [22, 26]. In addition, modification of sulphonyl‐based products has created a range of compounds, including sulphonyl nitriles, amides, and amines, that exhibit similar affinity and anti‐inflammatory effects when compared to the sulphonylurea‐based derivatives [10, 26, 27, 30]. The most prominently utilized molecules in preclinical models are OLT1177, YQ128, Bay 11‐7082, necrosulfonamide (NSA), JC124, and JC121 (Table 2) [20, 28, 31, 32]. Additionally, several molecules including NP3‐146, GDC‐2394, SN3‐1, Inzomelid, and ZYIL1 have entered phase 1/2 trials [33].

**TABLE 2:** Structures of modified Sulphonylurea and Sulphonyl based derivatives.

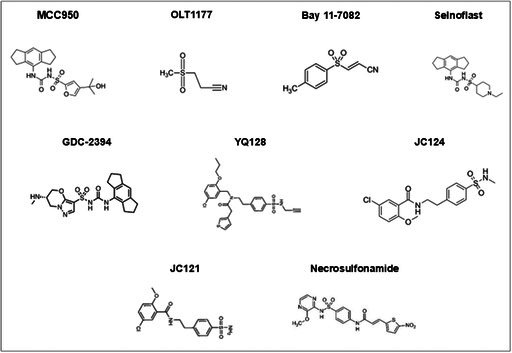

*Note:* The structures were adapted from [26, 27, 28].

## 3 The Role of NLRP3 Inflammasomes in Inflammatory and Autoimmune Conditions

### 3.1 Structure and Signaling of the NLRP3 Inflammasome

The NLRP3 inflammasomes are intracellular complexes that act as pattern recognition receptors (PRR) found within several immune cells, including monocytes, macrophages, and microglia [29, 34]. Structurally, the NLRP3 inflammasomes comprise several functional units that assist in their regulation, oligomerisation, and effector functions [35, 36]. The secreted inactive NLRP3 sensor consists of three domains: an amino‐terminal pyrin domain (PYD); a central nucleotide‐binding domain (NACHT); and a carboxy‐terminal leucine‐rich repeat (LRR) domain [35, 36]. The LRR and NACHT domains are responsible for the normal inhibition and self‐association functions [35]. The remaining units are the apoptosis‐associated speck‐like protein containing a caspase recruitment domain (ASC), which provides the interaction between the activated NLRP3 sensor and the pro‐caspase‐1 molecule [35]. Activation of the NLRP3 inflammasomes results in pyroptosis‐mediated cell death and the release of the activated pro‐inflammatory cytokines IL‐1β and IL‐18 that modulate the immune response to endogenous or pathogenic threats [9].

Primarily, NLRP3 activation occurs via the canonical pathway and requires two signals, the first of which involves PRR [9, 37]. Specifically, activation of the Toll‐like receptor (TLR)‐4 stimulates the inhibitory kappa kinase beta (IKKβ) pathway and translocation of the transcription factor nuclear factor kappa B (NF‐κB) (Figure 1) [9, 37]. In the nucleus, NF‐κB upregulates the production of the inactive NLRP3 sensor along with the secretion of pro‐IL‐1β and pro‐IL‐18 [9, 37]. The second signal is predominantly due to the increased presence of endogenous danger‐associated molecular patterns (DAMPs), although it may be induced by the presence of pathogen‐associated molecular patterns (PAMPs) via a noncanonical pathway [17]. This results in a broad range of sensor activators including extracellular ATP, low intracellular potassium levels, mitochondrial reactive oxygen species, lipopolysaccharides (LPS), and crystalline monosodium urate (MSU), some of which are a result of the primary autoimmune attack [9, 17, 37]. Following both signals, NLRP3 recruitment, oligomerization, and auto‐cleavage of the pro‐caspase‐1 occurs, releasing active caspase‐1 [9, 17]. Caspase‐1 then cleaves pro‐IL‐1β and pro‐IL‐18 into their active forms and triggers the formation of the gasdermin D (GSDMD) pore [9]. This pore has two primary functions: to induce pyroptosis and to act as a conduit for various ions and cytokine release including activated IL‐1β and IL‐18 [9, 38].

### 3.2 NLRP3 Products as Drivers of Autoimmune Conditions

The excessive production, activation, and release of IL‐1β, IL‐18, and GSDMD have been implicated in the development and progression of numerous autoimmune and inflammatory conditions, including RA, IBD, Parkinson's disease (PD), Alzheimer's disease (AD), and MS [9, 39, 40, 41]. The primary source of IL‐1β and IL‐18 for immunomodulatory and inflammatory responses is via the NLRP3 inflammasome pathway [10].

#### 3.2.1 IL‐1β

**FIGURE 2:**
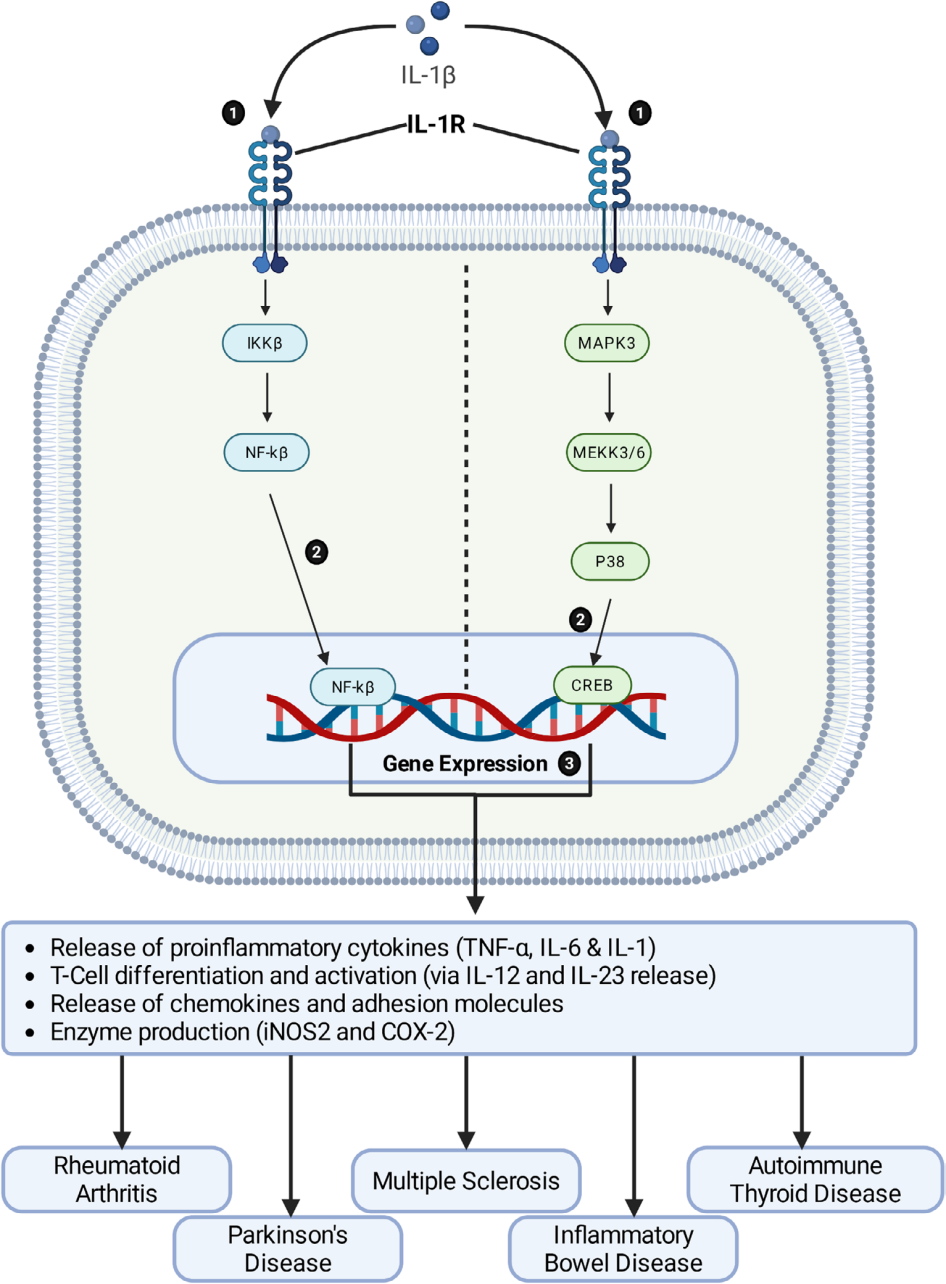
IL‐1β signaling cascade. In monocytes, macrophages, and B and T cells, activation of IL‐1R (1) by IL‐1β results in translocation of the transcription factors NF‐κB and CREB (2), which are responsible for the upregulation of multiple effector molecules associated with several autoimmune conditions (3). COX‐2, cyclooxygenase 2; CREB, cAMP‐response element binding protein; IKKβ, inhibitor of nuclear factor kappa B kinase subunit β; IL, interleukin; iNOS2, inducible nitric oxide 2; MAPK3, mitogen‐activated protein kinase 3; MEKK3/6, mitogen‐activated protein/ERK kinase kinase 3/6; NF‐κB, nuclear factor kappa B; p38, protein 38; TNF‐α, tumor necrosis factor‐alpha. Adapted from [41, 42] (Created with biorender.com).

In autoimmune conditions, IL‐1β exerts a range of inflammatory and immunomodulatory activities by affecting protein synthesis and intracellular energy production, as well as inducing B‐cell apoptosis and necrosis [40, 41]. It elicits cellular responses via the mitogen‐activated protein kinase (MAPK) signal transduction pathways that regulate several transcription factors, including NF‐κB, activator protein (AP)‐1, and CCAAT/enhancer‐binding protein (Figure 2) [29]. Therefore, IL‐1β activity in autoimmune conditions can have a wide range of disease‐dependent effects.

In RA, secretion of IL‐1β activates IL‐1 receptor subtype 2 (RII) on neutrophils, monocytes, B cells, and bone marrow cells, triggering the NF‐B signaling cascade to promote leukocyte infiltration, synovial hyperplasia, and joint destruction via the NF‐κB signaling cascade [9, 41, 43, 44]. In addition, activation of this induces the production of inflammatory cytokines, angiogenic chemokines, adhesion molecules, antiapoptotic factors, cell cycle regulators, and cell proliferative cytokines [9, 45].

By contrast, the pathogenesis of MS remains unclear; increased levels of myelin‐specific autoreactive T‐helper (Th)‐17 cells, along with IL‐17A and IL‐22 in the periphery and CNS, play a central role, with their activity reliant on an increased availability of IL‐1β [39, 46, 47, 48]. Additionally, IL‐1β has also been shown to promote dysregulation and destruction of CNS barriers that allow for infiltration of immune cells into the CNS, while NLRP3^−/−^ mice have demonstrated reduced T‐cell infiltration of the spinal cord and lymphoid tissue with milder disease outcomes [49].

Furthermore, in AD and PD, several increased markers within the NLRP3 signaling cascade suggest a role for autoimmune and inflammatory responses in the development and progression of both conditions [50, 51, 52, 53]. In AD, significant increases in cerebrospinal fluid concentrations of caspase‐1 and IL‐1β, along with elevated IL‐1β in the microglia surrounding β‐amyloid plaques, have been identified [54]. In PD, the formation of alpha‐synuclein aggregates leads to the chronic activation of TLRs in microglia, thereby increasing NLRP3 inflammasome activity and circulating IL‐1β levels [55, 56, 57, 58]. Additionally, ASC expression and elevated active caspase‐1 in postmortem brains from patients with PD illustrate the potential pathophysiological role of NLRP3 inflammasomes [57, 59, 60].

Finally, in IBD conditions such as Crohn's disease, it is hypothesized that chronic inflammatory signaling alters the innate immune response, leading to increases in Th‐2 cells in UC and Th‐1/Th‐17 cells [61, 62]. In addition, IL‐1β activity within the gastrointestinal tract also stimulates localized mucosal responses, increasing T and B cell proliferation and upregulating other pro‐inflammatory cytokines [62, 63]. NLRP3 inflammasome involvement in IBD has also been demonstrated by the overproduction of IL‐1β in disease models [62, 63].

#### 3.2.2 IL‐18

The role of IL‐18 in autoimmune and inflammatory diseases is less clear, although it is known to exert its effects via both innate and adaptive immune responses [42]. The most prominent pathway is in combination with IL‐12, to enhance natural killer (NK) cell activity [42, 64]. IL‐18 is also known to trigger interferon‐gamma (IFN‐γ) production from macrophages, which promotes the differentiation of T cells towards Th‐1 cells, while inhibiting Th‐2 and Th‐17 cells [42, 64]. Although there is less evidence of IL‐18 as a key driver of disease pathology, increased IL‐18 production has been demonstrated in both experimental models and patients with autoimmune conditions [42, 64].

In AD, IL‐18 promotes hyperphosphorylation of tau proteins via cyclin‐dependent kinase 5 (Cdk5) and glycogen synthase kinase‐3β (GSK‐3β), with the Cdk5 pathway also responsible for neuronal and mitochondrial fragmentation [65]. Although not fully elucidated, elevated levels of serum IL‐18 have also been demonstrated in patients with MS, IBD, psoriasis, and RA, which suggests a prominent role in disease development and progression [42]. In addition, IL18^−/−^ mice have shown better outcomes in collagen‐induced arthritis models, and by failing to develop experimental autoimmune encephalomyelitis (EAE) and autoimmune myasthenia gravis [66]. Overall, these observations support a role for IL‐18 in the pathogenesis of multiple conditions [66, 67, 68].

#### 3.2.3 GSDMD Pores

The role of GSDMD pore formation usually provides a pathway that functions to protect the host, although in chronic inflammatory diseases, it becomes an important gateway for the sustained immune response [69]. Primarily, pore formation leads to cell swelling and membrane lysis resulting in pyroptosis, protecting the host from intracellular infections [69]. Additionally, released pro‐inflammatory cytokines and intracellular material stimulate the innate and adaptive immune response to eliminate pathogenic threats [70, 71].

Highlighting the role of GSDMD pores in disease development are experimental models and human tissue samples. In experimental models, the regulation of GSDMD pore activity has demonstrated a reduction in neuroinflammation in EAE models, while pore formation is necessary for demyelination in MS [69, 70]. In models of psoriasis, GSDMD mediates the pathogenesis of the disease, while elevated levels of GSDMD have been identified in human psoriatic lesions [72, 73]. Additionally, patients with IBD also have significantly higher concentrations of GSDMD in tissue lesions, which drive the IL‐1β and IL‐18‐mediated disruption of epithelial permeability and mucosal barriers [73]. Although GSDMD may not directly induce the inflammatory response like proinflammatory cytokines, it plays a key role in the release of activated cytokines to the extracellular environment [69].

### 3.3 Targeting of NLRP3 by Sulphonyls

NLRP3 inflammasome signaling cascade has several regulatory points that can be targeted to disrupt the production and activation of the NLRP3 sensor, IL‐1β, IL‐18, and GSDMD pores (Figure 1). Glibenclamide, the most efficacious sulphonylurea currently available, inhibits inflammasome activity by preventing PAMP, DAMP, and crystalline activation of the NLRP3 sensor [11, 22]. In contrast, the most extensively studied sulphonylurea derivative is MCC950, which inhibits ATP hydrolysis and ASC oligomerization to suppress inflammasome activity [21, 74]. Selnoflast and GDC‐2394 share a similar R^2^ moiety to MCC950, and both are effective inhibitors of NLRP3 inflammasomes [19, 28]. GDC‐2394 exerts its effects by preventing ASC recruitment, while the mechanisms for selnoflast have yet to be elucidated [19, 28]. The β‐sulphonyl nitrile OLT1177 has shown similar functions to MCC950 by inhibiting ATPase activity and preventing recruitment of the NLRP3‐ASC CARD region, which is necessary for oligomerization [18, 20, 75]. OLT1177 also suppresses caspase‐1 activity, thereby limiting cytokine activation and release via the GSDMD pores [20, 75].

By contrast, JC121 and JC124 are sulphonyl amide derivatives of glibenclamide, and both cause selective inhibition of the NLRP3 inflammasome [21, 76]. Structurally, JC124 differs from JC121 due to the inclusion of a methylated amide, which results in mechanistic differences in the inhibitory process [77]. JC121 inhibits NLRP3‐ASC aggregation and caspase‐1 activity to reduce cytokine secretion and pyroptosis, whereas JC124 binds directly to NLRP3 without affecting the ATPase activity, suggesting a distinctive mode of action from the other sulphonyl derivatives [76, 77]. Further modification of the structure of JC124 resulted in the creation of YQ128, which significantly inhibits NLRP3‐mediated production of IL‐1β, but displays poor oral bioavailability [76]. Unlike other sulphonyls, Bay 11‐7082, has a broader effect as a strong inhibitor of IKKβ, preventing cytokine production and NLRP3 sensor production (Figure 1) [78]. Finally, necrosulfonamide, which was initially characterized by its ability to inhibit GSDMD pores, has also been shown to affect upstream pathways, reducing the activity of caspase‐1 and the activation of IL‐1β and IL‐18 [38, 71].

## 4 Sulphonyls in the Treatment of Autoimmune Conditions

The complexity of the development and progression of autoimmune conditions has resulted in limited therapeutic options for disease management. However, the off‐target interactions of sulphonylureas inhibiting various stages of the NLRP3 inflammatory cascade suggest a potential role as immunomodulatory agents. Therefore, evaluation of these therapeutic agents using preclinical models may provide critical insights into the therapy's direct impact on disease progression and their potential value as pharmacological interventions. NLRP3 targeted treatment in several preclinical models provides support for this approach (Table 3). Expanding upon these findings, an exploration of current treatment strategies and the use of sulphonyls in RA, MS, PD, and AD models merit individual consideration.

**TABLE 3:** In vivo studies using sulphonylureas and sulphonyls as anti‐inflammatory agents.

Model	Administration route	Drug, dosage, and timing	Inflammatory marker results
Parkinson's disease model 1‐methyl‐4‐phenyl‐1,2,3,6‐tetrahydro‐pyridine (MPTP)‐induced mouse model Male mice (C57BL/6)—8 weeks [52]	Intraperitoneal injection	MCC950–10 mg/kg—daily—for 13 days	↓ NLRP3 activity ↓ caspase‐1 ↓ IL‐1β maturation
Streptozocin‐induced diabetes model Mice (C57BL/6) [79]	Intraperitoneal injection	Glibenclamide—50 mg/kg—daily—starting 7 days before streptozocin injection	↓ IL‐1β No detectable changes in IFN‐γ, IL‐6, IL‐10 and TNF‐α
Parkinson's disease model paraquat and maneb‐induced mouse model Male mice (C57BL/6)—12 weeks [80]	Intraperitoneal injection	Glibenclamide—1 mg/kg—30 min before each paraquat and maneb injection (twice per week for 8 weeks)	↓ IL‐1β expression and maturation ↓ caspase‐1 ↓ NLRP3 expression ↓ reactive oxygen species
Experimental autoimmune encephalomyelitis (EAE) for multiple sclerosis (MS) Mice (C57BL/6) [81]	Intraperitoneal injection	JC‐171 (prophylactic)—100 mg/kg—on days 0, 1, and 2 post initial injection, and every second day thereafter JC‐171—10 mg/kg—every second day after clinical symptoms appeared MCC950–10 mg/kg—every second day after clinical symptoms appeared	↓ Th17 cells ↓ IL‐1β No detectable changes IL‐6 and TGF‐β
Collagen‐induced arthritis Mice (DBA/1 J)—8 weeks [82]	Intraperitoneal injection	MCC950–10 mg/kg—every second day for 2 weeks after clinical symptoms appeared	↓ NLRP3 expression ↓ IL‐1β ↓ caspase‐1 No detectable changes IL‐6 and TNF
Alzheimer's disease (AD) model Female mice (APP/PS1)—5 months [83]	Gavage (oral)	JC124–50 mg/kg and 100 mg/kg—daily (excluding weekends)	↓ microglia specific marker (Iba1) ↓cyclin D1—a marker of growth phase 1 of the cell cycle
Alzheimer's disease (AD) model Male mice (C57BL/6 and APP/PS1)—6 months [84]	Oral (feed pellets)	OLT1177–0, 3.75, or 7.5 g/kg—daily	↓ microglia activation ↓ plasma metabolic markers for AD
Highly refined carbohydrate (HC) diet model of cardiac hypertrophy and inflammation Male mice (Balb/c)—6 weeks [24]	Gavage (oral)	Glibenclamide—20 mg/kg—daily, from week 4 to 8 after commencement of HC diet	↓ caspase‐1
Experimental autoimmune encephalomyelitis (EAE) for multiple sclerosis (MS) Female mice (C57BL/6)—6 weeks [85]	Intraperitoneal injection	MCC950–10 mg/kg—days 0, 1 and 2 post initial injection, and every second day thereafter	↓ inducible nitric oxide synthase ↓ activation of astrocytes
Experimental autoimmune encephalomyelitis (EAE) for multiple sclerosis (MS) Female mice (C57BL/6)—4–6 weeks [86]	Gavage (oral)	MCC950–50 mg/kg—daily for 21 consecutive days commencing on day 16 post‐initial injection	No changes in plasma cytokines, but alleviated mechanical allodynia in the bilateral hind paws
Experimental autoimmune encephalomyelitis (EAE) for multiple sclerosis (MS) Female mice (C57BL/6)—8–10 weeks [20]	Intraperitoneal injection or gavage (oral)	OLT1177–200 mg/kg, 60 mg/kg (*i.p*.) and 60 mg/kg (oral)	↓ myelin loss ↓ spinal cord levels of IL‐18 and IL‐1β ↓ CD4+ T cells ↓ microglia ↓ macrophages
Osteoarthritis model Male mice (C57) – 7 weeks [87]	Injection (joint)	MCC950–3 mg/kg—prior to euthanasia	↓ NLRP3 expression ↓ IL‐1β
MSU crystal‐induced arthritis model Male mice (C57BL/6)—10–12 weeks [75]	Intraperitoneal injection or gavage (oral)	OLT1177–60, 200, or 600 mg/kg	↓ IL‐1β ↓ IL‐6 ↓ CXCL1

### 4.1 Rheumatoid Arthritis

Rheumatoid arthritis (RA) is a chronic inflammatory disorder wherein auto‐antibodies lead to the degradation of synovial joints [88]. Currently, first‐line treatment for RA involves methotrexate (MTX), a disease‐modifying anti‐rheumatoid drug [89]. Although there is much debate regarding the mechanism by which MTX mediates its anti‐inflammatory effects in this condition [90]. Treatment with MTX results in the suppression of T‐cell activation, downregulation of B cells, and inhibition of IL‐1β binding to its cell receptor [89]. Preclinical models of RA utilizing sulphonylurea derivatives as a therapeutic agent have demonstrated that inhibition of NLRP3 also results in several positive disease outcomes [75, 91, 92]. In particular, oral treatment with OLT1177 (≥ 60 mg/kg) in murine models of MSU crystal‐induced gouty arthritis resulted in a dose‐dependent reduction of joint swelling, reduced IL‐1β production, and reduced leukocyte infiltration [75]. In addition, synovial hyperplasia and cartilage erosion were impeded in collagen‐induced arthritis and osteoarthritis (OA) murine models treated with MCC950 (3 mg/kg and 10 mg/kg) and JC121 (10 mg/kg) [82, 87]. Although positive results were achieved, other RA disease markers, such as IL‐6, were not modified with sulphonyl treatment; therefore, disease progression still occurred but at a slower rate compared to control groups [75, 91, 92]. Considering the association of IL‐1β with increased leukocyte infiltration, angiogenesis, and synovial proliferation, the supplementary use of NLRP3 inhibitors could further improve RA symptoms and reinforce existing treatment protocols.

### 4.2 Multiple Sclerosis

Multiple sclerosis is an autoimmune and neurodegenerative disease characterized by demyelination and eventual loss of axon function in the CNS [47]. In MS, treatment with interferon‐β products aims to reduce the stimulation and polarization of the CD4^+^ T cells into Th‐1 and Th‐17 cells via the disruption of the antigen‐presenting cells [48, 93]. To support current treatments, sulphonyls such as OLT1177 (60 mg/kg) and MMC950 (50 mg/kg/day) provided protective effects in EAE murine models, reducing functional decay, preventing relapses, and improving histological outcomes [20, 86]. Furthermore, MCC950 (10 mg/kg) and JC171 (10 mg/kg) treatments have also reduced serum levels of IL‐1β and attenuated spinal cord demyelination [81, 85, 94]. Therefore, as a complementary treatment, NLRP3 inhibitors may further inhibit IL‐1β, IL‐18, caspase‐1, and GSDMD pore activity, thereby reducing cytotoxic T cell activity and increasing the polarization of T cells into T regulatory cells [48, 95].

### 4.3 Alzheimer's Disease

Alzheimer's disease is considered a chronic autoimmune condition in which the development of β‐amyloid plaques and neurofibrillary tau tangles (NFTs) results in synaptic loss and neuronal atrophy primarily throughout the cerebral cortex and hippocampus [96, 97, 98]. Currently, no treatments exist that effectively target tau aggregation and neurotoxicity; therefore, mitigation of symptoms and delaying cognitive decline are the primary focuses [52]. Two drug classes are regularly utilized, namely acetylcholinesterase inhibitors and N‐methyl D‐aspartate glutamate receptor antagonists, which augment acetylcholine longevity in the synapse to increase cognitive function and reduce the neurotoxicity associated with glutamate hyperexcitability, respectively [99]. Given the lack of effective treatments to address disease progression in AD, the development of agents that inhibit NLRP3 may provide additional support to delay this process. This is evidenced by improved cognitive function and reduced accumulation of β‐amyloid plaques in mice treated with OLT1177 (0, 3.75, or 7.5 mg/kg) and JC124 (50 mg/kg or 100 mg/kg) [83, 84]. Indirect inhibition of the NLRP3 inflammasomes by the fenamate class of nonsteroidal anti‐inflammatory drugs, such as flufenamic acid (20 mg/kg), is also associated with a reduction in IL‐1β expression and microglial activation, leading to subsequent reductions in neuroinflammation [100]. Furthermore, treatment via caspase‐1 inhibition by VX‐765 (25 mg/kg) is effective in reversing neuroinflammation and normalizing inflammatory markers and cognitive impairment [101]. Therefore, the sulphonyls may present a new avenue to attenuate disease progression in AD, providing therapeutic support to a condition lacking a targeted therapy.

### 4.4 Parkinson's Disease

Parkinson's disease is characterized by the reduction of dopamine levels caused by dopaminergic degeneration in the substantia nigra (SN), while accumulation of misfolded protein aggregates forming Lewy bodies is also commonly observed [102, 103]. Although PD is a neurodegenerative condition, there is evidence to suggest a role for autoimmune and inflammatory responses in the development and progression of the disease [50, 51]. Given the complexity of PD and the absence of disease‐modifying treatments, current therapeutic approaches aim to counteract dopamine loss [56, 58]. Primary PD therapies either increase production or reduce degradation of dopamine through the use of the dopamine precursor levodopa (L‐dihydroxyphenylalanine), dopamine receptor agonists, and monoamine oxidase B inhibitors, as well as promotion of presynaptic dopamine release with dopamine reuptake inhibitors (amantadine) and catechol‐O‐methyl transferase inhibitors [104].

As a potential alternative, treatment of PD murine models with glibenclamide (1 mg/kg) resulted in significantly reduced alpha‐synuclein upregulation, dopaminergic neurodegeneration, and motor defects [80]. In addition, MCC950 (10 mg/kg) improved behavioral impairments and reduced neuronal degeneration in the 1‐methy‐4‐phenyl‐1,2,3,6‐tertrahydropyridine (MPTP) induced PD mouse model [52]. Treatment of the same PD models utilizing glibenclamide (1 mg/kg) and necrosulfonamide (0.5 mg/kg or 1 mg/kg) also reduced disease progression and presentation of clinical symptoms [31, 52].

## 5 Key Findings and Future Directions

Overall, the sulphonylureas and their derivatives have demonstrated therapeutic efficacy in modulating the development and progression of several autoimmune‐mediated inflammatory conditions in experimental models. While the use of sulphonylureas in the treatment of autoimmune conditions may be restricted due to the hypoglycaemic risk, doses less than those that induce hypoglycaemia appear to attenuate inflammatory processes and disease progression. Consequently, sulphonylureas could serve as a beneficial treatment option for several inflammatory and autoimmune conditions. Additionally, modifying the specificity and duality of glibenclamide could lead to the development of molecules that control comorbidities commonly associated with autoimmune conditions. Furthermore, the use of derivatives such as MCC950, selnoflast, JC124, and OLT1177 targeting the NLRP3 inflammasome has yielded noteworthy results, which have attenuated the disease development and progression in models of PD, RA, IBD, and MS. Although several promising candidates have entered phase 2 clinical trials, there may be a role for repurposing currently available substances such as glibenclamide until other therapeutic agents are approved for clinical use.

Finally, there is an unexplored opportunity for this drug class to act as adjunct therapies. By targeting multiple pathways in autoimmune conditions, they could provide greater control over symptoms and disease progression. To date, no studies have compared the NLRP3 inhibition against or in conjunction with current treatment protocols. Therefore, to better predict patient outcomes and ascertain the long‐term efficacy of these compounds, it is necessary to include these comparisons in future models.

## Nomenclature of Targets and Ligands

Key protein targets and ligands in this article are hyperlinked to corresponding entries in http://www.guidetopharmacology.org, the common portal for data from the IUPHAR/BPS Guide to PHARMACOLOGY [105], and are permanently archived in the Concise Guide to PHARMACOLOGY 2019/20 [106].

## Author Contributions

**Jasen Elford:** conceptualization, data curation, writing – original draft, writing – review and editing. **Robert J. O'Reilly:** conceptualization, writing – review and editing. **Anna‐Marie Babey:** writing – review and editing. **Natkunam Ketheesan:** conceptualization, funding acquisition, project administration, supervision, writing – review and editing. **Rukshan Ahamed Mohamed Rafeek:** conceptualization, project administration, supervision, writing – review and editing.

## Conflicts of Interest

The authors declare no conflicts of interest.

## Data Availability

Data sharing is not applicable to this review article as no new data were created or analyzed in this study.
